# The perceived impact of family physicians on the district health system in South Africa: a cross-sectional survey

**DOI:** 10.1186/s12875-018-0710-0

**Published:** 2018-02-05

**Authors:** Klaus B. von Pressentin, Robert J. Mash, Laurel Baldwin-Ragaven, Roelf Petrus Gerhardus Botha, Indiran Govender, Wilhelm Johannes Steinberg, Tonya M. Esterhuizen

**Affiliations:** 1 Division of Family Medicine and Primary Care, Faculty of Medicine and Health Sciences, Stellenbosch University, PO Box 241, Cape Town, 8000 South Africa; 2 Department of Family Medicine, School of Clinical Medicine, University of the Witwatersrand, Johannesburg, South Africa; 3 Department of Family Medicine, University of Pretoria, Pretoria, South Africa; 4 Family Medicine, Sefako Makgatho Health Sciences University, Pretoria, South Africa; 5 Family Medicine, University of the Free State, Bloemfontein, South Africa; 6 Biostatistics Unit, Centre for Evidence-based Health Care, Department of Global Health, Stellenbosch University, Cape Town, South Africa

**Keywords:** Family physicians, District health system, Perceived impact, Primary health care, Cross-sectional study

## Abstract

**Background:**

Evidence from first world contexts support the notion that strong primary health care teams contain family physicians (FPs). African leaders are looking for evidence from their own context. The roles and scope of practice of FPs are also contextually defined. The South African family medicine discipline has agreed on six roles. These roles were incorporated into a family physician impact assessment tool, previously validated in the Western Cape Province.

**Methods:**

A cross-sectional study design was used to assess the perceived impact of family physicians across seven South African provinces. All FPs working in the district health system (DHS) of these seven provinces were invited to participate. Sixteen respondents (including the FP) per enrolled FP were asked to complete the validated 360-degree assessment tool.

**Results:**

A total number of 52 FPs enrolled for the survey (a response rate of 56.5%) with a total number of 542 respondents. The mean number of respondents per FP was 10.4 (SD = 3.9). The perceived impact made by FPs was high for five of the six roles. Co-workers rated their FP’s impact across all six roles as higher, compared to the other doctors at the same facility. The perceived beneficial impact was experienced equally across the whole study setting, with no significant differences when comparing location (rural vs. metropolitan), facility type or training model (graduation before and ≥ 2011).

**Conclusions:**

The findings support the need to increase the deployment of family physicians in the DHS and to increase the number being trained as per the national position paper.

**Electronic supplementary material:**

The online version of this article (10.1186/s12875-018-0710-0) contains supplementary material, which is available to authorized users.

## Background

Strong primary health care (PHC) systems lead to better health outcomes for the population they serve [1, 2]. The 2008 World Health Report “Primary Health Care - Now More Than Ever” defines strong PHC systems as those systems which offer first contact care that is patient-centred with an orientation to the patient’s family and community context, embedded in a service that is comprehensive, integrated, continuous, and community-orientated, and in which patient-care is well co-ordinated [1]. This report warned against oversimplified approaches to PHC in developing countries, which only focus on priority diseases or rely on unsupported health workers who are poorly equipped for the complexity of PHC. The World Health Assembly supports the report’s recommendation that PHC should be offered by a multidisciplinary team that includes a family physician (FP) [3–6].

The contribution of family medicine to health system improvement has been supported by empirical research evidence, policy documents and public statements from global health leaders [7–11]. The research evidence base, however, arises mainly from high income settings, in which communities and health systems are different from their LMIC (low and middle income country) counterparts [12].

Defining the roles of the family physician (or general practitioner) with postgraduate training has been a complex undertaking since the origins of academic family medicine in the 1960’s [13–16]. The golden thread remains the commitment to the individual within the context of their family and community. The roles of the FP are based on values and principles that are shared almost universally [11, 17]. However the roles and scope of practice are also contextually defined by factors such as whether the FP is the first contact person, whether the FP also provides hospital based care and the skills-mix in the PHC team [11, 18].

In the African LMIC region, healthcare is characterised by a significant imbalance between the high burden of disease and the scarcity of healthcare workers to address this burden, especially doctors, nurses and midwives [19–21]. Family medicine only obtained recognition in the past two decades, with South Africa obtaining family medicine “specialty status” in 2007 [22]. The 2009 WONCA (World Organisation of Family Doctors) Africa regional conference reached a consensus statement on family medicine in Africa, describing the role of the FP as “a clinical leader and consultant in the primary health care team, ensuring primary, continuing, comprehensive, holistic and personalized care of high quality to individuals, families and communities.” This consensus document forms the core understanding and basis for further development of family medicine in Africa [23].

**Fig. 1:**
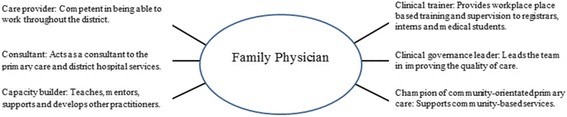
The six key roles of the South African family physician [22]

In South Africa, a recent national position paper summarised the local consensus on the roles and competencies required of FPs [22]. This paper represents the viewpoint of the South African Academy of Family Physicians (SAAFP) and the College of Family Physicians of South Africa (CFPSA), and articulates the potential contribution of FPs to district health services within the national policy framework. Key elements of this policy framework include the National Development Plan, policy on Human Resources for Health and draft legislation on health system re-engineering towards establishing universal health coverage and national health insurance [24–26]. Six key roles for the FP were described (Fig. 1) [22], which link closely with regional and international trends:

- Care provider: a competent clinician, able to deal with the majority of the health problems in the community that he or she serves, within the district hospital or primary care setting, and competent in relational skills (including patient centeredness, communication skills and bio-psychosocial approach).
- Consultant: as part of a well-functioning healthcare team, he or she provides support to other practitioners (e.g. clinical nurse practitioners and medical officers) by seeing referred patients in primary care facilities or district hospitals.
- Capacity builder: as a senior clinician, he or she is responsible for the mentoring and training of less qualified clinicians within the primary health care or district hospital teams.
- Clinical trainer: he or she may need to function as the clinical supervisor in the workplace for medical students, clinical associate trainees, interns or registrars.
- Clinical governance leader: responsible for improving the quality of clinical services within the sub-district and facility where he or she is appointed.
- Champion of community-orientated primary care (COPC): supports the development of a COPC approach to the district health system, particularly the development and integration of ward-based outreach teams (a team of community health workers responsible for a geographically defined group of households).

In South Africa policymakers and managers of the health services continue to hold diverse views on the value of FPs and provinces have significant autonomy when deciding whether to employ FPs at scale [22, 27]. Although the entry of FPs into the healthcare system is relatively recent, evidence of their impact would contribute to decision making. Prior to 2007 FPs were trained in a variety of part-time programmes, whereas after 2007 FPs were trained in full-time registrar posts to a set of national learning outcomes and started entering the health system from 2011. Within the African region evidence of their impact would also be of value to countries that are contemplating the training of FPs or deciding on whether to scale up training [28]. The African continent remains the only continent that has not widely embraced the training of FPs [12].

In order to collect evidence of the early impact of FPs in South Africa a national research project utilised four different methods – a quasi-experimental study, a survey of FPs using a validated 360-degree impact assessment tool, correlation of FP supply with routine district health indicators and interviews with district managers. This article presents the findings from the survey and the other studies will be reported elsewhere. Using a validated 360-degree impact assessment tool [29], this study aimed to evaluate the impact of FPs within the district health services of South Africa from the perspective of those working around them at district hospitals or primary care facilities. The study also intended to compare their perceived impact with that of medical officers who had not received postgraduate training. Secondary outcomes include comparing the perceived impact of FPs between rural and metropolitan (urban) contexts, by facility type (district hospitals and community health centres), and training programme model (graduation before and after 2011).

## Methods

### Study design

A cross-sectional survey of the perceived impact of FPs was conducted using a validated 360-degree FP impact assessment tool [29]. The STROBE (Strengthening the Reporting of Observational Studies in Epidemiology) checklist for cross-sectional studies was used to guide this report [30].

### Setting

The study was conducted in public sector district health services (DHS) within seven out of the nine provinces of South Africa, as determined by the provincial footprint of the six participating universities.

FPs in the public sector are primarily employed within the DHS, and are typically based at a district hospital (DH), community health centre (CHC) or sub-district and provide support to the sub-district’s PHC services. Some FPs are also based at the regional hospital (within a family medicine, emergency medicine or out-patient department) or at the level of the district, either as a district FP with a general clinical governance role or a more specific role for maternal and child health care as a member of a District Clinical Specialist Team (DCST) [22].

### Characteristics and selection of participants

All FPs working in the DHS (public sector) of these seven provinces were invited to participate (via the collaborating academic family medicine departments, who consulted their departmental databases of all the DHS-employed FPs). FPs working at regional or tertiary hospitals, those working only at the district level (DCST or district office) or those working in the private sector, were excluded as their scope of practice did not encompass that of the six agreed FP roles as described in the national position paper [22].

After accepting the invitation, providing their written informed consent and receiving a briefing on the data collection procedure, each FP generated a list of 25–30 potential respondents from within their work environment (this list included the co-workers’ job titles and contact information). This list of potential respondents was converted by the lead researcher into an Excel document and sorted into three categories: people who the FP reports to (e.g. managers), people who work alongside the FP (e.g. senior medical officers, pharmacists) and people who report to the FP (e.g. junior medical officers, nurses). For each participating FP, five respondents were randomly selected from each of these categories. The final list of participants contained the study codes of the FP (self-assessment) and his/her 15 selected co-workers (sixteen respondents in total).

### Data collection tool

This study made use of a Family Physician Impact Assessment Tool, which was developed, validated and piloted previously in the Western Cape Province of South Africa [29]. The tool comprises of a section that provides information on use of the tool, a section that gathers information about the respondents and a section that asks about six domains representing each of the six previously described FP roles (Fig. 1).

Each domain consisted of a number of positively-phrased statements as items, with six response options in a Likert scale format: not performed by the FP (0), strongly disagree (1), disagree (2), agree (3) and strongly agree (4). Respondents could also state that they were not able to answer that question.

One additional item was added for each domain as a whole, asking the respondents to compare the impact of the FP to that of the medical officers at the same facility. This additional item had seven Likert scale options: does not perform this role (0), significantly less impact (1); less impact (2); same impact (3); more impact; (4), significantly more impact (5); and unable to answer.

Respondents were asked to complete the tool either on a hard copy (printed booklet) or online via a secure webpage (Stellenbosch University: Checkbox® survey software version 6) [31].

### Data collection process

The research team included local co-ordinators from each of the academic family medicine departments and a total number of 16 fieldworkers were trained. A fieldwork protocol ensured a uniform approach to conducting the fieldwork. The fieldwork team contacted the FP and each respondent (in person, via email or phone call) and invited them to complete the paper or online version of the tool. The completed paper tools were collected in a secure box in a neutral space within the facility or collected in person by the fieldwork team. Data were collected between March 2015 and February 2016. Data collected on paper were captured with EpiData version 3.1 [32] via a double-entry method and using checks to minimize data entry errors. Answers to the individual statements were substituted for numbers according to the Likert scale. The data captured online were exported to Excel as a SPSS compatible file. The data from the two versions were combined into a single database. This database was checked for any errors and protected.

### Data analysis

Data analysis was performed using IBM SPSS version 23 [33] and a data analysis protocol. Descriptive analysis was performed to describe the FPs in terms of the number of respondents, provincial distribution, location (metropolitan vs. rural), DHS facility type (district hospital vs. community health centre) and training model (qualification as FP before 2011 and ≥ 2011).

Data analysis of the perceived impact for each domain or role was conducted at the level of the FP (the unit of analysis), which required aggregating the respondent-level data into domain mean values. The data analysis protocol specified that no score per domain at individual respondent level will be calculated into the total mean score at the level of the FP, should more than 50% of the items (questions) in a domain not be answered.

A weighted mean score for each FP role was calculated based on the Likert scale from 0 to 4. The mean score was weighted according to the number of respondents per FP as the assessment would be more valid with more respondents. The weighted mean score could then be interpreted as:

- Score < 1.5: No impact in this area
- Score ≥ 1.5 but < 2.5: Little impact in this area
- Score ≥ 2.5 but < 3: Moderate impact in this area
- Score ≥ 3: High impact in this area

A comparison was made between the various roles to assess if the perceived impact of the FPs differed between the roles. A repeated measures analysis of variance (ANOVA) was used to obtain an overall *p*-value (Wilks’ Lambda test statistic) for the differences between the six roles. Subsequently, paired samples t-test analyses were performed to compare the six individual roles against each other (15 pairs in total were compared). Using the Bonferroni correction, the level of α (critical p-value) was divided by the number of tests [34]. The new α for comparing the weighted means between the individual domains was calculated by dividing 0.05 with 15, which resulted in a new critical p-value of 0.0033 for the between-role comparisons.

The FP’s perceived impact in each role was compared to the medical officers. The score for the comparative evaluation between FPs and the other medical officers (Likert scale from 0 to 5) was interpreted as follows:

- Score < 2.5: In favour of the medical officers for a particular role
- Score ≥ 2.5 but < 3.5: No perceived difference between medical officers and the FP for a particular role
- Score ≥ 3.5: In favour of the FP for a particular role

The independent samples test (t-test for equality of means) was used to assess the relationship between the mean scores for each domain and the secondary outcomes (metropolitan vs. rural, district health facility type, qualification before and after 2011).

### Ethical considerations

This study was approved by the Health Research Ethics Committee, Stellenbosch University (reference S15/01/003), as well as the relevant institutions and provincial authorities in the study setting (the full list is available as an Additional file 1). All respondents gave their written informed consent and study codes were used to enter the information into the anonymised database.

## Results

A total number of 52 FPs enrolled for the survey with a total number of 542 respondents. The average response rate of the eligible, invited FPs was 56.5% (see Table 1) and the mean number of respondents per FP was 10.4 (SD = 3.9; minimum 1 and maximum 18), which indicated a response rate of 63.9% (542/848) for the invited respondents.

**Table 1:** Family physician and respondent enrolment per province

Province	Family physicians employed in DHS at facility level		Total number of respondents per province	
	Invited and eligible	Enrolled	Invited	Enrolled
Free State	4	3	48	48
Gauteng	28	12	192	107
KwaZulu-Natal	8	6	96	76
Mpumalanga	12	5	80	53
North West	5	5	80	71
Northern Cape	2	2	32	24
Western Cape	33	19	304	163
Total	92	52	848	542

The distribution of enrolled FPs by rural vs. metropolitan location, facility type and training programme was fairly equal (Table 2). The enrolment by province was unequally distributed. Table 3 outlines the four different groups of respondents who evaluated the enrolled FPs. Only 30 of the 52 enrolled FPs (57.7%) completed the tool as a self-assessment. An additional 14 FPs participated as respondents for other FPs, which meant that of all the respondents, 44 (8.1%) were FPs.

**Table 2:** Distribution of enrolled FPs by DHS facility type, rural/metropolitan location and training programme type

Rural/Metropolitan			FP training programme		Total
			New	Previous	
Rural	Facility type	CHC	5	6	11
		DH	9	9	18
	Total		14	15	29
Metro	Facility type	CHC	3	11	14
		DH	6	3	9
	Total		9	14	23
Total	Facility type	CHC	8	17	25
		DH	15	12	27
	Total		23	29	52

*CHC* community health centres, *DH* district hospitals

**Table 3:** Profile of respondents (*N* = 542)

Respondent Category	n (%)
Managers (including district, facility and operational managers)	111 (20.5)
Colleagues (including other family physicians, senior medical officers, allied health workers and pharmacists)	169 (31.2)
Junior colleagues (including family medicine registrars and junior doctors) and colleagues who consult the family physician (including nurses and community health workers)	164 (30.3)
Family physician self-evaluations	30 (5.5)
Undefined role (including missing data for this variable)	68 (12.5)
Total	542 (100.0)

**Fig. 2:**
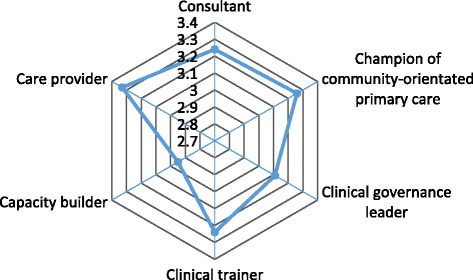
The overall perceived impact of the participating family physicians across the seven provinces (weighted means)

Table 4 presents the weighted and unweighted mean values for each domain and demonstrates a close correlation. The FP was seen to have a high impact in all six roles apart from that of capacity builder, which was rated as a moderate impact. The perceived impact for the role of capacity builder was significantly (*p* < 0.001) lower compared to the other roles. The remaining between role-comparisons were not significantly different. The mean values for each role are visually represented in the radar graph (Fig. 2). FPs were also perceived to have a greater impact than the medical officers across all six roles with a mean score above 3.5 (Table 5). No significant differences were found when comparing the weighted means according to rural vs. metropolitan location, facility type (DH vs. CHC), and the training model (Table 6).

**Table 4:** Mean values per domain (family physician role)

Domain	Unweighted means		Weighted means	
	N	mean (SD)	N	mean (SD)
Care provider	52	3.31 (0.36)	52	3.33 (1.26)
Consultant	52	3.23 (0.36)	52	3.24 (1.25)
Capacity builder	52	2.93 (0.36)	52	2.95 (1.16)
Clinical trainer	52	3.21 (0.37)	52	3.24 (1.30)
Clinical governance leader	51	3.13 (0.63)	51	3.11 (1.29)
Champion of community-orientated primary care	52	3.25 (0.36)	52	3.26 (1.30)

**Table 5:** Perceived impact in each role compared to the other doctors at the facility

Additional question for each domain	Unweighted means	Weighted means
	Mean (SD)	Mean (SD)
Care provider	3.91 (0.65)	3.87 (1.54)
Consultant	3.92 (0.63)	3.87 (1.55)
Capacity builder	3.63 (0.60)	3.61 (1.47)
Clinical trainer	3.89 (0.72)	3.86 (1.59)
Clinical governance leader	3.91 (0.87)	3.82 (1.62)
Champion of community-orientated primary care	3.99 (0.58)	3.94 (1.54)

*A mean value greater than 3.5 is in favour of the family physician making a greater impact than the other doctors at the facility for this role (Likert scale 0–5)

**Table 6:** Comparison of weighted means for secondary outcomes

Domain		N	Mean (SD)	t	*p*-value
Rural vs. metropolitan location					
Care provider	rural	29	3.39 (1.13)	0.441	0.661
	metro	23	3.24 (1.42)		
Consultant	rural	29	3.31 (1.16)	0.468	0.642
	metro	23	3.15 (1.38)		
Capacity builder	rural	29	2.99 (1.12)	0.286	0.776
	metro	23	2.90 (1.23)		
Clinical trainer	rural	29	3.27 (1.19)	0.183	0.856
	metro	23	3.20 (1.46)		
Clinical governance leader	rural	28	3.17 (1.30)	0.365	0.717
	metro	23	3.04 (1.29)		
Champion of community-orientated primary care	rural	29	3.31 (1.18)	0.289	0.774
	metro	23	3.21 (1.45)		
DHS facility type					
Care provider	CHC	25	3.27 (1.14)	− 0.285	0.777
	DH	27	3.37 (1.38)		
Consultant	CHC	25	3.22 (1.12)	− 0.129	0.898
	DH	27	3.26 (1.38)		
Capacity builder	CHC	25	2.97 (1.04)	0.096	0.924
	DH	27	2.94 (1.28)		
Clinical trainer	CHC	25	3.26 (1.15)	0.109	0.914
	DH	27	3.22 (1.45)		
Clinical governance leader	CHC	24	3.26 (1.18)	0.776	0.441
	DH	27	2.98 (1.38)		
Champion of community-orientated primary care	CHC	25	3.27 (1.15)	0.043	0.965
	DH	27	3.26 (1.44)		
New vs. previous training model					
Care provider	new	23	3.27 (1.23)	− 0.284	0.778
	previous	29	3.37 (1.30)		
Consultant	new	23	3.11 (1.22)	− 0.650	0.519
	previous	29	3.34 (1.29)		
Capacity builder	new	23	2.74 (1.10)	−1.208	0.233
	previous	29	3.13 (1.19)		
Clinical trainer	new	23	3.07 (1.27)	− 0.842	0.404
	previous	29	3.37 (1.33)		
Clinical governance leader	new	22	2.77 (1.17)	− 1.664	0.102
	previous	29	3.37 (1.33)		
Champion of community-orientated primary care	new	23	3.08 (1.24)	− 0.914	0.365
	previous	29	3.41 (1.34)		

*significant at *p* < 0.05

## Discussion

### Summary

The impact made by family physicians in DHS facilities across seven South African provinces was perceived by co-workers as high for five of the six agreed roles: care provider, consultant, clinical trainer, leader of clinical governance and champion of community-orientated primary care. Their impact in the role of capacity builder was significantly less than the other roles although still seen as a moderate impact. Furthermore, co-workers rated their FP’s impact across these six roles as higher than the other doctors at the same facility. The perceived beneficial impact was experienced equally across the whole study setting, as no significant differences were found when comparing location, facility type or training model.

### Comparison with existing literature

#### Overall perceived impact for the different roles

The role of the FP in COPC was perceived to be much higher in the national study compared to the initial pilot study. This may be due to the greater involvement of family medicine departments in the implementation of COPC in provinces such as Gauteng and the limited commitment of the Western Cape, where the pilot study was conducted, to implement COPC as part of PHC re-engineering.

Conversely, the role of the FP in building the capacity of the healthcare team was rated much lower in the national study compared to the pilot study. This might be due to the emphasis on implementing clinical governance in the districts included in the pilot study [35]. In other provinces the DCSTs may have taken more of a lead in capacity building around maternal and child health [36]. FPs in the Western Cape may also have had a greater emphasis on educational skills in their training programme [37].

A new postgraduate training programme was introduced post-2007 aimed at equipping the new graduates with the competencies needed for their six roles. This study, however, did not demonstrate a significant difference in perceived impact between FPs graduating from the old and new programmes. This may be attributed to the many years of vocational experience of FPs trained previously that ensured they developed the competencies required for their roles [22]. In addition, newly qualified FPs may require some time to grow into all six roles, especially the leadership roles, which require a degree of seniority and maturity [22].

#### Comparison of their impact relative to the medical officers

An ongoing concern voiced by managers in the health system revolves around the perceived benefit of employing a FP with postgraduate training vs. a MO with a lower salary package [22, 38, 39]. This study found that FP co-workers (including the facility managers who function closer to the FP’s circle of control and influence) rated their perceived impact higher compared to the other doctors within the facility for all six roles.

#### Relationship between their impact in these roles and other indicators of health system performance and clinical processes

The study was not designed to demonstrate a causative link or correlation between the six FP roles and health outcomes. Nevertheless the six roles are incorporated by the DOH into the job description of FPs as these roles are important to strengthening the DHS and national health priorities [24, 26]. If policy makers agree on the potential value embodied by these six roles, further investment in the training and employment of FPs within the DHS should be promoted.

The link between FP supply and health system indicators has been explored and an ecological study did not show a demonstrable correlation from the macro-perspective of the district, as the FP supply was still too low [40]. A cross-sectional study, however, demonstrated how FPs add value to in-hospital clinical care (especially in terms of paediatric care) [41]. Qualitative studies have also explored this question from the perspective of the district managers and suggested that family physicians have had a positive impact on the quality of clinical processes across the quadruple burden of diseases, and some impact on health services performance (in terms of improved access to care, better coordination, and the provision of a more comprehensive and efficient service) [22, 38, 42].

### Implications for research and practice

Given the alignment of these six roles with national priorities for strengthening the DHS and the perceived impact of FPs demonstrated in this study, there is a need to employ FPs at scale and to strengthen the training programmes (including number of registrar posts) as described in the national position paper [22].

The finding of a lower perceived impact in the capacity building role may require more research to understand the factors involved, such as the implementation of clinical governance in districts or training provided to registrars for this role.

The Family Physician Impact Assessment tool could be used to evaluate the perceived impact of FPs in other African or LMIC health systems, providing that the FPs are similarly positioned within the PHC teams and share the same roles. This will require adaptation and validation of the instrument for the new context, but may produce useful data for between country-comparisons.

This study should be repeated after 5-years in order to evaluate changing perceptions of the impact of FPs as more FPs are employed and the health system undergoes further transformation.

### Limitations

This study was conducted congruent to the methods described in the pilot study, by conducting the analysis at the level of the FP (and not at the level of the individual respondent) [29]. The weighting of means to control for the variation in respondent numbers per FP resulted in larger standard deviations, which decreased the likelihood of finding statistically significant findings when comparing sub-groups. The weighting was applied in the direction of FPs with more respondents, as this increased the number of observations per FP which should result in a more valid 360-degree appraisal [43].

The potential bias of asking the FPs to nominate respondents was addressed in the pilot study’s article [29]. The researchers implemented the pilot study’s recommendation of selecting respondents randomly from the total pool of eligible people in each category [29]. Further bias may be introduced by only considering responses from FPs (and their co-workers) who chose to enrol for this study (this enrolment bias may have excluded better and/or worse performing FPs). The enrolment procedure included several strategies to help convince eligible FPs of the social and scientific value of the study, and inform them of the measures employed to ensure anonymity. Fortunately, Table 2 demonstrates the equal distribution of enrolled FPs by location, facility type and training model. As the tool included responses from the FPs themselves a sub-analysis was performed to see if excluding the self-assessments would alter the results. This sub-analysis demonstrated that the same results were obtained for the six domains as well as for the comparison of the FP’s perceived impact to that of the other medical officers. The unequal distribution by province was predetermined by the differences in employment of eligible FPs [39]. The exclusion of two South African provinces (Eastern Cape and Limpopo) was predetermined by the provincial footprint of the academic family medicine departments. The number of eligible FPs in these excluded provinces was small and their omission from the survey is unlikely to have a substantial effect on the findings.

## Conclusions

FPs working within the DHS have a high perceived impact in their roles as care providers, consultants, clinical trainers, leaders of clinical governance and champions of community-orientated primary care, and a moderate perceived impact as capacity builders. Their impact was perceived to be greater than the medical officers at the same facilities across all six roles. The impact was the same regardless of location, facility type or training model. The findings support the need to increase the deployment of family physicians in the DHS and to increase the number being trained as per the national position paper.

## Additional file

Additional file 1: Full list of HREC approvals and PHRC/DRC permissions (DOCX 16 kb)

## Abbreviations

ANOVA: Analysis of variance
CFP(SA): The College of Family Physicians of South Africa
CHC: Community health centre
COPC: Community-orientated primary care
DCST: District clinical specialist team
DH: District hospital
DHS: District health system
FP: Family physician
LMIC: Low and middle income country
MO: Medical officer
PHC: Primary health care
SAAFP: South African Academy of Family Physicians
SD: Standard deviation
STROBE: Strengthening the Reporting of Observational Studies in Epidemiology
WHO: World Health Organization
WONCA: World Organisation of Family Doctors
