# Supplementary material for: The perceived impact of family physicians on the district health system in South Africa: a cross-sectional survey
Source: BMC Fam Pract. 2018 Feb 5;19:24. doi: 10.1186/s12875-018-0710-0 (PMC5800021; doi:10.1186/s12875-018-0710-0)
Supplement: Additional file 1: — Full list of HREC approvals and PHRC/DRC permissions (DOCX 16 kb) [file 12875_2018_710_MOESM1_ESM.docx]

**Full list of HREC approvals and PHRC/DRC permissions**

| Health Research Ethics Committees | Reference number |
| --- | --- |
| Stellenbosch University | S15/01/003 |
| University of KwaZulu-Natal | S15/01/003 |
| University of the Free State | ECUFS 28/2015 |
| University of the Witwatersrand | M150488 |
| Sefako Makgatho Health Sciences University | S15/01/003 |
| University of Pretoria | Ref 95/2015 |
| Provincial Health Research Committees | **Reference number** |
| Western Cape | WC_2015RP19_867 |
| KwaZulu-Natal | HRKM 034/15; KZ_2015RP21_947 |
| Free State | dated 22 May 2015 |
| Northern Cape | NC2015RP11168 |
| Gauteng | GP_2015RP12_549 |
| North West | NW_2015RP16_816 |
| Mpumalanga | MP_2015RP43_146 |
| Johannesburg District Research Council | 2015-16/007 |
| Tshwane Research Council | 52/2015 |

HREC: Health Research Ethics Committee

PHRC: Provincial Health Research Committee

DRC: District Research Committee
